# A Case of Autoimmune Small Fiber Neuropathy as Possible Post COVID Sequelae

**DOI:** 10.3390/ijerph20064918

**Published:** 2023-03-10

**Authors:** Noel G. Panagiotides, Fritz Zimprich, Klaus Machold, Oliver Schlager, Markus Müller, Sebastian Ertl, Henriette Löffler-Stastka, Renate Koppensteiner, Patricia P. Wadowski

**Affiliations:** 1 Division of Angiology, Department of Internal Medicine II, Medical University of Vienna, 1090 Vienna, Austria; noel.panagiotides@meduniwien.ac.at (N.G.P.); oliver.schlager@meduniwien.ac.at (O.S.); markus.a.mueller@meduniwien.ac.at (M.M.); sebastian.ertl@meduniwien.ac.at (S.E.); renate.koppensteiner@meduniwien.ac.at (R.K.); 2 Division of Cardiology, Department of Internal Medicine II, Medical University of Vienna, 1090 Vienna, Austria; 3 Department of Neurology, Medical University of Vienna, 1090 Vienna, Austria; friedrich.zimprich@meduniwien.ac.at; 4 Division of Rheumatology, Department of Internal Medicine III, Medical University of Vienna, 1090 Vienna, Austria; klaus.machold@meduniwien.ac.at; 5 Division of Internal Medicine II, Klinikum Wels-Grieskirchen, 4600 Wels-Grieskirchen, Austria; 6 Department of Psychoanalysis and Psychotherapy, Medical University of Vienna, 1090 Vienna, Austria; henriette.loeffler-stastka@meduniwien.ac.at

**Keywords:** autoimmune small fiber neuropathy, SARS-CoV-2, long COVID, primary Sjögren’s syndrome

## Abstract

Severe acute respiratory syndrome coronavirus 2 (SARS-CoV-2) infection is reported to induce and augment autoimmune processes. Moreover, postinfectious effects of coronavirus disease 2019 (COVID-19) are still poorly understood and often resemble symptoms of the acute infection phase. A patient with swollen extremities was presented to the Department of Angiology at the Medical University of Vienna with complaints of muscle and joint pain, paresthesia, and arterial hypertension with intense headache. Prior to these complaints, she had been suffering from various symptoms since November 2020, following a SARS-CoV-2 infection in the same month. These included recurrent sore throat, heartburn, dizziness, and headache. Paresthesia and muscle and joint pain started in temporal relation to a human papillomavirus (HPV) vaccination. Since the patient was suffering from severe pain, intensive pain management was performed. Skin and nerve biopsies revealed autoimmune small fiber neuropathy. The patient’s condition could be related to COVID-19, as her first symptoms began in temporal relation to the SARS-CoV-2 infection. Furthermore, in the disease course, antinuclear (ANA) and anti-Ro antibodies, as well as anti-cyclic citrullinated peptide (anti-CCP) antibodies, could be detected. Together with the symptoms of xerophthalmia and pharyngeal dryness, primary Sjögren’s syndrome was diagnosed. In conclusion, though biopsy results could not distinguish a cause of the disease, SARS-CoV-2 infection can be discussed as a likely trigger for the patient’s autoimmune reactions.

## 1. Introduction

Damage to the peripheral nerves may result in small fiber neuropathy (SFN) [1], affecting the thinly myelinated Aδ-fibres and unmyelinated C-fibres [2]. In addition, somatic and autonomic fibers, responsible for thermal and pain perception and the control of autonomic and enteric functions, can be affected, resulting in either autonomic or somatic symptoms or both [3]. Clinical patients may present with a myriad of symptoms which may involve allodynia, burning sensations, reduced thermal sensations, hyperesthesia, paresthesia, numbness in lower extremities, restless leg syndrome, dry eyes and mouth, abnormal perspiration, problems with bladder control, gastric issues, skin discoloration, and cardiac symptoms such as syncope, palpitations, and orthostatic hypotension [4].

The leading cause of SFN is diabetes mellitus, followed by autoimmune disorders, sarcoidosis, paraproteinemia, and paraneoplastic syndrome, but it remains unknown in about half of the cases [1,5,6]. In addition, genetic variations such as mutated transthyretin (TTR) lead to inherited amyloid polyneuropathies [7]. Sodium channel dysfunction due to the sodium voltage-gated channel alpha subunit (SCN) 9A or SCN11A mutation has also been linked to the development of SFN [8,9,10].

In addition to patient examination and history, nerve conduction studies, and, particularly, skin biopsies, as well as quantitative sensory testing are performed to diagnose SFN [1]. Additional tests, including measuring small fiber-related evoked potentials corneal confocal microscopy, and genetic testing, may also prove helpful [1,3,11].

In general, the therapy consists of the treatment of the underlying cause, if it can be identified [4], pain management, and other symptomatic therapies [12,13].

SARS-CoV-2 infection has recently emerged as a global burden comprising various complications affecting mainly the respiratory and cardiovascular system [14,15]. Viral infection is limited by an intact endothelial surface layer and causes subsequent inflammation/endotheliitis and thrombosis [16,17,18,19]. Moreover, SARS-CoV-2 infection is reported to trigger autoimmune disorders [20]. In this context, vasculitis and rheumatologic diseases are frequent. In the reported cases, vasculitis of the skin was most common (above 60% of the cases), followed by vasculitis of the kidneys, eyes, lymph nodes, heart, lungs, joints, and the aorta [20]. The gastrointestinal system, the liver, the ear-nose-throat region, brain, spleen, temporal, and iliac arteries were less frequently affected (below 10%) [20].

In addition, the occurrence of rheumatoid arthritis, spondylarthritis, systemic lupus erythematodes, reactive arthritis, and inflammatory myopathies may be triggered [20]. On the other hand, primary Sjögren’s syndrome can be associated with the development of severe peripheral neuropathy, which might even lead to limb weakness [21]. The development of Sjögren’s syndrome itself, however, is complex, and various factors including viral infections are discussed as a potential trigger [22,23].

Many patients continue to experience various symptoms after recovery from acute SARS-CoV-2 infection, commonly referred to as “long COVID” [19,24]. These symptoms can include cardiac, respiratory, neurological, musculoskeletal, nasopharyngeal, and psychological disorders [24]. Similar to our case, the complaints may consist of chest pain, joint and muscle pain, neuralgia, neuropathy, paraesthesia, headaches, tinnitus, dizziness, and sore throat [25,26,27,28,29,30,31]. Complications can arise within months after SARS-CoV-2 infection [32].

Postinfectious autonomic impairment includes lightheadedness, orthostatic headache, syncope, hyperhidrosis, and burning pain [32]. Moreover, the occurrence of autonomic dysfunction is often related to neuropathy [33]. Impaired sympathetic skin responses of patients with persistent pain, myalgia, or muscle cramps after COVID-19 were shown to be associated with SFN [34]. 

## 2. Case Presentation

**Figure 1:**
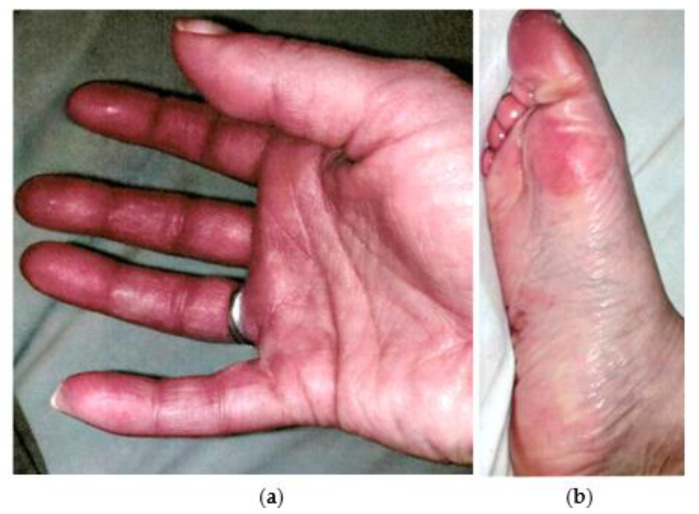
Pictures of hands and feet at admission. The patient showed swelling and redness of the right palm and fingers (**a**), as well as the sole of the right foot and toes (**b**).

A 34-year-old woman presented in November 2021 to the Department of Angiology at the Medical University of Vienna with severe pain as well as redness and diffuse swelling of the hands and feet. In addition, muscle and joint pain, as well as paresthesia, occurred. The symptoms emerged in March 2021 a few days after human papillomavirus (HPV) vaccination. Her symptoms began with muscle pain and muscle twitching that lasted a few seconds to about 2 min, resulting in pain, swelling, and redness in both hands and feet (Figure 1), which worsened with exposure to heat. After regular activity, the patient also described occasional paresthesia in both hands, which required her to hold her hands in an elevated position to relieve symptoms. Additionally, the patient suffered from occasional tinnitus and dizziness, as well as arterial hypertension with strong headaches and visual impairment due to vitreous detachments that required hospitalization in August 2021. The symptoms left her mostly bedridden and in severe pain most of the time. Prior to these complaints, she had been suffering from various symptoms since November 2020, following a SARS-CoV-2 infection in the same month. These included blood pressure problems, tachycardia, dizziness, blurred vision, general weakness, recurrent sore throat, heartburn, headache, and various gynecologic symptoms due to endometriosis. Her preexisting conditions included cervical syndrome, Hashimoto’s thyroiditis since 2012, diplopia and blurred vision in the left eye after a flu-like infection in 2016, and endometriosis since 2017. Prior to admission to the Medical University of Vienna, the patient consulted several physicians and hospitals and underwent numerous diagnostic tests including a detailed rheumatologic examination, which revealed elevated anti-myeloperoxidase antibodies (194.8 UA/mL) and anti-Scl-34 antibodies. However, there was no neurologic testing and the patient received no causative diagnosis.

## 3. Materials and Methods

The patient was admitted to the General Hospital of Vienna (Medical University of Vienna) for further evaluation.

### 3.1. Medical History

Upon arrival, a medical history was taken, and a thorough physical examination was performed. Interdisciplinary co-operation included among others rheumatologic, dermatologic, and neurologic councils.

The patient disease course was followed up via telephone contact and using the clinical database of the General Hospital of Vienna.

### 3.2. Laboratory Testing

#### 3.2.1. Routine and Rheumatologic Parameters

Laboratory tests comprising routine and rheumatologic parameters were obtained. The latter consisted of antinuclear antibody measurements including also anti-Ro-52 antibody measurements. Ro-52 mediates inflammatory responses and anti-Ro-52 antibodies have been associated with diverse autoimmune diseases, viral infections, and neoplastic diseases [35,36,37].

#### 3.2.2. SCN9A Gene

Sequential analysis of alterations in the SCN9A gene was performed from EDTA-blood. Genetic changes in the SCN9A gene are related to a number of conditions including erythromelalgia, congenital insensitivity to pain, paroxysmal extreme pain disorder, small fiber neuropathy, genetic epilepsy with febrile seizures plus, and hereditary sensory and autonomic neuropathy type 2 [8,9,38,39].

#### 3.2.3. SARS-CoV-2 Testing

SARS-CoV-2 polymerase chain reaction (PCR) tests and anti-SARS-CoV-2 antibody immunoassays were routinely performed at the Department of Laboratory Medicine of the Medical University of Vienna. According to the hospital regulations, SARS-CoV-2 PCR tests were performed via nasal swab.

For the determination of the antibody response, the qualitative Elecsys^®^ Anti-SARS-CoV-2 immunoassay (Roche Diagnostics, Penzberg, Germany), which detects antibodies against the nucleocapsid antigen of SARS-CoV-2, and the quantitative Elecsys^®^ Anti-SARS-CoV-2 S immunoassay (Roche Diagnostics, Penzberg, Germany), which detects antibodies against the receptor-binding domain of the SARS-CoV-2 spike protein, were used [40,41,42]. 

The patient received preparations of 20 g and 10 g immunoglobulin (Privigen), both produced in the year 2021 (April and June 2021, respectively) by CSL Behring (King of Prussia, PA, USA) five days before SARS-CoV-2 antibody testing.

### 3.3. Noninvasive Diagnostics

Noninvasive vascular studies included the obtainment of the ankle–brachial index (ABI) [43], pneumatic segmental pulse oscillography, optical oscillography [44,45], as well as duplex sonography of the temporal arteries and supraaortic branches. Furthermore, echocardiography and renal artery ultrasound were performed. The microcirculation was evaluated via nailfold capillary microscopy. 

### 3.4. Dermatologic and Neurologic Testing

Dermatologic testing, including patch testing, was performed based on the skin abnormalities. Next, neurological testing, including nerve conduction velocity and neurocranium imaging via MRI, was conducted. Following these tests, a skin biopsy (immunohistochemical analyses), a nerve biopsy (histologic/immunohistochemical analyses, semi-thin section, electron microscopy), autonomic tests, as well as tilt table testing was performed. Additional genetic testing was done.

#### 3.4.1. Nerve Conduction Studies

As an addition to an extensive neurological examination, nerve conduction studies were performed to evaluate the integrity and function of the peripheral nerves, including nerve conduction and impulse propagation of motor and sensory fibers. Via an external electrical impulse, an action potential was generated, transmitted, and recorded. Nerve responses, which indicate the number of functioning axons and the speed of the fastest nerve fibers, were recorded and compared to normative, physiological values. Significant differences can be interpreted as axon loss or demyelination, among others, and help identify nerve pathophysiology [46]. 

#### 3.4.2. Nerve Biopsy

The sural nerve is usually used for nerve biopsy due to accessibility. It is purely sensory and contains 5 to 10 nerve fascicles. These fascicles are myelinated as well as unmyelinated nerve fibers. Regarding the fact that one side is more affected than the other in some diseases, the more damaged nerve is usually used for harvest.

After extraction, the unfixed nerve specimen was divided into three preparations. Fixation was performed in a 4% phosphate-buffered formalin solution, 3.9% phosphate-buffered glutaraldehyde, and the third part was frozen and cut with a cryostat.

The paraffin-embedded tissue was used for standard histological stains and immunohistochemistry, glutaraldehyde fixed tissue for light microscopy or electron microscopy, fresh frozen unfixed tissue for histology and histochemistry, as well as protein, DNA, and RNA molecular pathology and genetic analysis [47].

## 4. Results

### 4.1. General Clinical Findings

Physical examination revealed redness and swelling on both hands and feet (Figure 1) with no further abnormalities. Likewise, extensive laboratory blood testing, including screening for infectious diseases, showed no abnormal results. During the hospital stay, the patient developed new efflorescences. Dermatologic examination revealed findings consistent with dyshidrotic eczema and spontaneous urticaria. In addition, allergic testing found an allergy to disperse red dye. 

Tilt table testing revealed a significant increase in heart rate (above 30 beats per minute) after a change in the table to the almost vertical (70°) position, indicating postural orthostatic tachycardia syndrome (POTS). The patient did not experience syncope or significant changes in blood pressure. 

### 4.2. Laboratory Findings

#### 4.2.1. Routine and Rheumatologic Parameters

On admission, differential blood count and electrolytes were without abnormalities. Routine laboratory analyses such as creatinine (0,73 mg/dL), ASAT (19 U/L), GGT (21 U/L), CK (87 U/L), NT-proBNP (75 pg/mL), CRP (0.1 mg/dL), and HbA1c (4.9%) were unremarkable. Several laboratory values were not within the normal range—cholesterol levels (total cholesterol 249 mg/dL, triglycerides 179 mg/dL, lipoprotein(a) 141 nmol/L, low-density lipoprotein 150.2 mg/dL, high-density lipoprotein 63 mg/dL) and serum TSH (4.44 µLU/mL) were elevated.

Antinuclear antibodies (ANA) including extractable nuclear antigen (ENA) subsets were negative at the initial testing at the Medical University of Vienna. However, perinuclear anti-neutrophil cytoplasmic antibodies (p-ANCA) were slightly elevated (10 IU/mL). In the further patient course, however, ANA titers turned positive with 1:80 and anti-Ro-antibodies were 39 U/mL. Furthermore, anti-cyclic citrullinated peptide (anti-CCP) antibodies were 7.5 U/mL. In addition, the myositis-associated antibody against Ro-52 was positive. 

With the symptoms of xerophthalmia (pathological Schirmer’s test), as well as pharyngeal dryness, the diagnosis of a primary Sjögren’s syndrome was given [48].

#### 4.2.2. Genetic Testing

There were no signs of disease-associated alterations in the SCN9A gene and, therefore, no signs of SCN9A-related erythromelalgia in genetic testing.

#### 4.2.3. SARS-CoV-2 Testing

Routine SARS-CoV-2 PCR testing via nasal swabs was negative during the entire stay. Anti-SARS-CoV-2 antibodies against the nucleocapsid protein were positive. Anti-SARS-CoV-2-S antibodies were increased with 693.00 U/mL (reference value is <0.80 U/mL). By detection of anti-SARS-CoV-2 antibodies against the spike protein, the occurrence of antibodies against the nucleocapsid protein could be confirmed. This indicates an existing or expired infection with the SARS-CoV-2 virus.

### 4.3. Noninvasive Diagnostics

#### 4.3.1. Sonography and Oscillography

**Figure 2:**
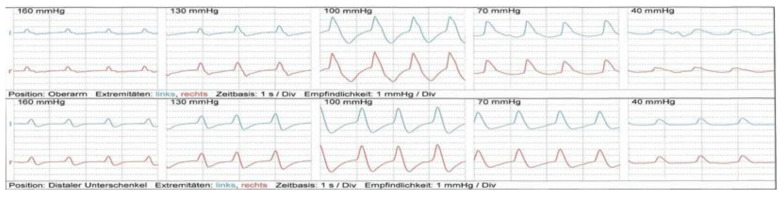
Pneumatic segmental pulse oscillography (P-SPO) of upper and lower extremities at rest. Pneumatic segmental pulsoscillography (P-SPO) of upper and lower extremities at rest showed regular findings. Upper half: upper arm, blue = left arm; red = right arm. Lower half: distal lower leg, blue = left leg; red = right leg; l = left; r = right.

**Figure 3:**
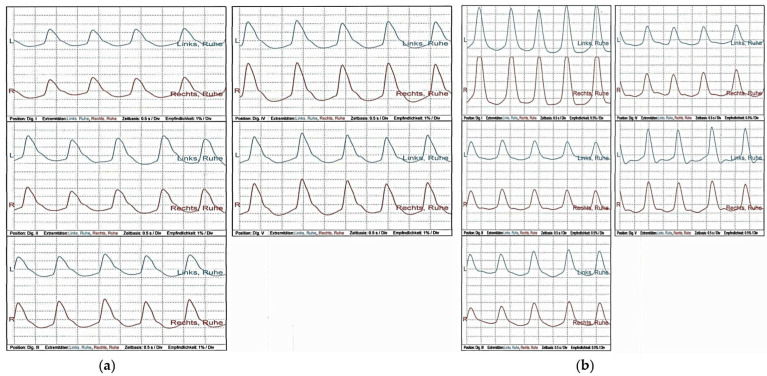
Optical pulse oscillography of all fingers (**a**) and toes (**b**) at rest. Optical pulse oscillography of all fingers and toes showed regular findings. Left (**a**): Fingers I–V, blue = left hand; red = right hand. Right (**b**): Toes I–V, blue = left foot; red = right foot.

Color-coded duplex sonography of the supraaortic arteries, superficial temporal artery, and subclavian artery revealed no evidence of vasculitis. In addition, oscillography and distal arterial pressures yielded no evidence of macroangiopathy (Figure 2 and Figure 3). The ABI was regular. Echocardiography showed no pathologies.

Renal vasculature assessed by color-coded duplex sonography showed no abnormalities. Sonography of the kidneys and urinary tract revealed regular findings. The kidneys appeared normal in size with a longitudinal diameter of 10 cm on both sides. Parenchymal and sinus echoes were inconspicuous with no evidence of hydronephrosis. There was no evidence of a mass in the suspected area of the adrenal gland.

#### 4.3.2. Capillary Microscopy

**Figure 4:**
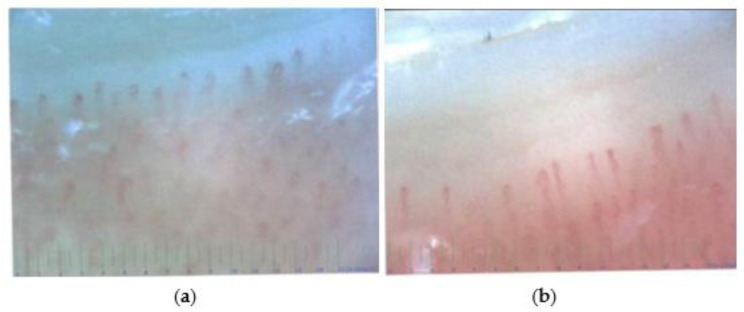
Capillary microscopy at the nailfold. Nailfold microscopy showed mostly regular capillaries with occasional non-pathological dilatations, regular density, and regular blood flow. Thus, microangiopathy could not be detected. Left side (**a**): left digit V. Right side (**b**): right digit IV.

Capillary microscopy showed no evidence of microangiopathy at the nailfold (Figure 4). 

### 4.4. Dermatologic and Neurologic Testing

#### 4.4.1. Skin Biopsy

The patient’s skin biopsies revealed a significant reduction in intraepidermal fiber density in the sample from the lower leg as well as the one from the thigh. The value in the lower leg was below the lower 5th percentile threshold of the reference value for women between 30 and 39 years of age. The combined reduction in intraepidermal nerve fiber density in the lower leg and thigh compared with the reference values could also indicate “non-length-dependent” SFN.

#### 4.4.2. Nerve Conduction Studies

A nerve conduction velocity was ordered based on the symptoms, which yielded pathological findings after stimulation of the N. median and tibial nerve, compatible with SFN. A skin biopsy (see Section 4.4.1.), a serological determination of immune-mediated neuropathies, and a nerve biopsy (see Section 4.4.3.) were performed to confirm the diagnosis.

Sensory neurography revealed no anomalies in all examined nerves. 

Motor neurography showed prolonged distal motor latency (DML), markedly reduced compound muscle action potential (CMAP) amplitude and slowed nerve conduction velocity (NCV) over the entire course of the right peroneal nerve; a slightly prolonged DML and reduced CMAP amplitude were also seen over the left peroneal nerve.

These findings were compatible with a primary axonal motor lesion of the peroneal nerve and axonal polyneuropathy (PNP). Criteria for immune-mediated PNP such as chronic inflammatory demyelinating polyneuropathy (CIDP) or Guillain-Barre syndrome (GBS) were not met.

The sympathetic skin response at the upper and lower extremities after median nerve stimulation at the wrist and the tibial nerve at the ankle was pathological, with response potentials having low amplitudes and markedly prolonged potentials, which served as evidence of autonomic nerve fiber degeneration. 

These changes were beyond SFN alone.

#### 4.4.3. Nerve Biopsy

In the nerve biopsy of the right sural nerve a pronounced reduction in the unmyelinated fibers, and a slight reduction in the myelinated fibers was observed. There were no signs of inflammatory processes, de-, or remyelination. Further, there was no fiber deterioration or regeneration present. Endoneurial vessels showed no wall thickening. Altogether, the findings were compatible with the results of the skin biopsy. 

## 5. Therapeutic Measures and Outcomes

Initially, the patient’s pain was managed with gabapentin and paracetamol. The dermatological findings resulted in the treatment of the skin with topical corticosteroids in addition to general skincare measures, as well as antihistamine treatment (levocetirizine). In addition to previous medications (levothyroxine for Hashimoto’s thyroiditis), angiotensin converting enzyme (ACE) inhibitor therapy for hypertension and ivabradine therapy for autonomic neuropathy with recurrent tachycardia were started. To manage POTS, the patient was informed about possible non-pharmacologic measures such as compression stockings, drinking amount, and pharmacologic measures such as low-dose beta-blockers.

Assuming a possible autoimmunological neuropathy with concomitant small fiber and autonomic neuropathy, short-term steroid therapy was attempted but remained without effect.

Intravenous therapy with immunoglobulin was initiated and repeated at regular intervals. Therapy was well tolerated by the patient, but resulted only in a modest improvement in neuropathic pain and intermittent redness of the hands. 

In the further disease course, the nonspecific complaints persisted, which included muscle weakness, paraesthesia, dry eyes and mouth, fatigue, impaired circulation in the hands, palpitations, and orthostatic symptoms. 

Since symptoms only slightly improved, it was decided to discontinue immunoglobulin therapy after six cycles and to start hydroxychloroquine therapy. Due to the suspected association with SARS-CoV-2 infection, the patient was also included in the local long-COVID registry. At the patient’s request, further therapy took place at the local hospital in Salzburg, since the patient did not live in Vienna. 

After a few months, the disease course was followed up via telephone contact. The patient reported that her condition had improved dramatically following immunoglobulin therapy relative to before, however, hydroxychloroquine was not well tolerated, and symptoms worsened again. Hydroxychloroquine was discontinued, intravenous immunoglobulin therapy was resumed, and rituximab and low-dose cortisone were added. The patient added that, in retrospect, immunoglobulin therapy and high-dose cortisone improved her condition, but low-dose cortisone and hydroxychloroquine did not.

## 6. Discussion

The patient was diagnosed with autoimmune SFN. The obtained histopathologic changes were beyond SFN alone, possibly augmented by the patient’s comorbidities such as the autoimmune disorders Sjögren’s syndrome and Hashimoto’s thyroiditis [3,21,49]. However, one of the main causes of SFN is diabetes [1,50], which was not present in our patient.

After pain management and immunoglobulin therapy, the patient’s quality of life improved significantly, however, the improvement lasted only for a short period. The disease may be temporally related to the SARS-CoV-2 infection, as the first symptoms appeared after the COVID-19 infection and anti-SARS-CoV-2 antibodies were increased. Moreover, as viral persistence after COVID-19 occurs [51], evoking post COVID sequelae, the patient’s symptoms may be part of a long COVID syndrome. 

Patients frequently experience various symptoms after recovering from acute SARS-CoV-2 infection [19,24]. Furthermore, there has been a growing number of reports linking SFN with SARS-CoV-2 infection and “long COVID” [52,53,54,55], indicating SFN as a potential factor contributing to the paresthesia experienced by post-COVID-19 patients [52]. Currently, infection-triggered immune dysregulation leading to autonomic dysfunction is discussed as the main underlying cause [53]. Herein, inflammatory processes may promote the development of neuritis [53]. Moreover, the binding of SARS-CoV-2 to neurons of the dorsal root ganglion, which express the mRNA of SARS-CoV-2 (associated) receptors including angiotensin-converting enzyme 2 (ACE2) mRNA and protein, is discussed. [53,56,57]. This process might be considered as the viral entry side affecting the sensory nervous system and evoking persistent symptoms [56,57]. However, further research is needed to understand the exact mechanisms through which COVID-19 infection may be related to SFN.

The disease course of the presented patient was possibly augmented by HPV vaccination, after which additional symptoms manifested. A study on individuals, who developed a chronic disease soon after HPV vaccination found that musculoskeletal pain, fatigue, headache, dizziness or vertigo, paresthesia or allodynia, and nausea or vomiting were the most common complaints after HPV vaccination, with a high likelihood of experiencing neuropathic pain [58]. Autonomic dysfunction is discussed as a rare side effect of HPV vaccinations; however, causality could not be proven up to date [59,60]. One trigger discussed for the development of small fiber disease after HPV vaccination is neuroinflammation [59], which was not present in the nerve biopsy of our patient.

Adverse reactions to HPV vaccination such as hypersensitivity or urticarial vasculitis have been reported [61,62]; however, true hypersensitivity reactions are uncommon and most with suspected hypersensitivity to vaccines tolerate revaccination [61,63]. 

While in the hospital, the patient presented with new skin rashes. Upon examination by a dermatologist, the symptoms were consistent with dyshidrotic eczema and spontaneous urticaria and received topical corticosteroids and antihistaminic medication, which improved her condition.

In addition, the development and progression of SFN might also be enhanced by hypothyroidism and the primary Sjögren’s syndrome [3,21,49].

Therapy of SFN is often difficult and requires interdisciplinary collaboration for establishing an adequate therapy regimen and adherence to therapy [64]. The disease burden, coupled with uncertainty of disease cause and therapeutic effectiveness, is known to evoke psychological and socioeconomic consequences. Effective containment and working through uncertainties would be necessary within the doctor-patient relationship in order to (re-) establish a good long-lasting quality of life and a sustainable treatment regimen. The containment of anxieties needs a secure framework and precise patient-centered work on the individual level [65,66]. 

On the systems level, interdisciplinary efforts [67] and effective patient involvement [68] is necessary for good patient-centered health-care management. 

One major limitation of our case report is the administration of the intravenous immunoglobulin preparation five days before anti-SARS-CoV-2 antibody measurement. There is increasing evidence that anti-SARS-CoV-2 antibodies are present in currently available immunoglobulin products [69,70,71]. Therefore, an anti-SARS-CoV-2 antibody measurement might be biased in the reported patient. However, the COVID-19 infection was confirmed by SARS-CoV-2 PCR testing. Viral persistence evoking the described symptoms—possibly augmented by the HPV vaccination—might be anticipated. One further limitation is that the direct reactions to the HPV vaccination were not investigated but rely on the patient’s anamnesis. 

## 7. Conclusions

In conclusion, autoimmune processes known to be triggered by SARS-CoV-2 infection are a likely cause of SFN [20,53]. Though the development of SFN might also be due to the primary Sjögren’s syndrome, at least a multifactorial disease etiology can be anticipated. Our case report is intended to raise awareness to clinicians for correctly diagnosing SFN and to reevaluate ANA and ENA subsets in the patient disease course to obtain a correct diagnosis and document new, possible complications after SARS-CoV-2 infection.

## Data Availability

Anonymized data presented in this case report are available on request from the corresponding author.
